# Transformation of Coherent Twin Boundary into Basal-Prismatic Boundary in HCP-Ti: A Molecular Dynamics Study

**DOI:** 10.3390/ma17092165

**Published:** 2024-05-06

**Authors:** Tao Sun, Qili Bao, Yang Gao, Shujun Li, Jianping Li, Hao Wang

**Affiliations:** 1 State Key Laboratory of Rolling and Automation, Northeastern University, Shenyang 110819, China; gaoyang@ral.neu.edu.cn (Y.G.); ljp@mail.neu.edu.cn (J.L.); 2 Institute of Metal Research, Chinese Academy of Sciences, Shenyang 110016, Chinashjli@imr.ac.cn (S.L.); 3 Interdisciplinary Centre for Additive Manufacturing (ICAM), School of Materials and Chemistry, University of Shanghai for Science and Technology, Shanghai 200093, China

**Keywords:** titanium, grain boundary, dislocation, plastic deformation, molecular dynamics

## Abstract

The manufacturing process for wrought Ti alloys with the hexagonal close-packed (HCP) structure introduces a complicated microstructure with abundant intra- and inter-grain boundaries, which greatly influence performance. In the hexagonal close-packed (HCP) structure, two types of grain boundaries are commonly observed between grains with ~90° misorientation: the basal/prismatic boundary (BPB) and the coherent twin boundary (CTB). The mechanical response of the BPB and CTB under external loading was studied through molecular dynamic simulations of HCP-Ti. The results revealed that CTB undergoes transformation into BPB through the accumulation of twin boundary (TB) steps and subsequent emission of Shockley partial dislocations. When the total mismatch vector is close to the Burgers vector of a Shockley partial dislocation, BPB emits partial dislocations and further grows along the stacking faults. When a pair of CTBs are close to each other, severe boundary distortion occurs, facilitating the emission and absorption of partial dislocations, which further assists the CTB-BPB transformation. The present results thus help to explain the frequent observation of coexisting CTB and BPB in HCP alloys and further contribute to the understanding of their microstructure and property regulation.

## 1. Introduction

Titanium and its alloys are widely used in various industries, including aerospace, aeronautics, surgery and dental, chemical, and nautical, due to their outstanding mechanical, biomechanical, and anti-corrosion properties [1]. However, the manufacturing process for wrought Ti alloys introduces a complicated microstructure with abundant intra- and inter-grain boundaries, which greatly influence performance. Understanding the transformation between different boundaries is crucial for revealing the formation mechanism of various microstructures and clarifying the microstructure–property relationship.

In the hexagonal close-packed (HCP) structure, two special high-angle grain boundaries (GB) that have garnered increasing attention due to their coexistence are the $<1\overset{-}{2}10>\{1\overset{-}{1}02\}$ coherent twin boundary (CTB) [2] and the basal-prismatic boundary (BPB) [3]. The CTB has twinning elements K and, with a misorientation angle of ~90° between the matrix and twin, e.g., 84.5° and 86.2° for Ti (c/a = 1.58) and Mg (c/a = 1.62), respectively. The BPB has basal and prismatic orientations on each side with coherency along $\left[1\overset{-}{2}10\right]$ and the misorientation angle is exactly 90° for all HCP systems. Due to their orientation-wise similarity, the CTB and BPB are theoretically believed to be closely related. In fact, the BPB has been observed in many HCP metals, such as Mg [4,5], Zn [6], and Co [5,7], and almost all BPBs are found with abundant $\{1\overset{-}{1}02\}$ CTBs nearby. Both simulation and experimental results show that the BPB plays an important role during the nucleation and growth of $\{1\overset{-}{1}02\}$ CTB [8,9,10].

Regarding HCP-Ti, despite being energetically less favored than Mg or other HCP metals [11,12,13], the 90°-related boundary appears to be more relevant for certain special mechanical behaviors. Regarding the dwell fatigue problem [14], grains with a misorientation larger than 80° have always been found in samples with a dwell crack [15], where high-angle GBs are considered to cause significant stress concentration [16,17,18]. Since we only know the rough grain misorientation rather than the exact boundary orientation, there are many possibilities regarding local plastic deformation [19]. Therefore, in this study, we revisit $\{1\overset{-}{1}02\}$ CTB and BPB on the atomic scale with molecular dynamics simulation and concentrate on the transformation behaviors of these two boundaries in HCP-Ti, aiming at further clarifying their relationship and providing theoretical support for investigating the fatigue behaviors of HCP-Ti and its alloys.

## 2. Method

**Figure 1:**
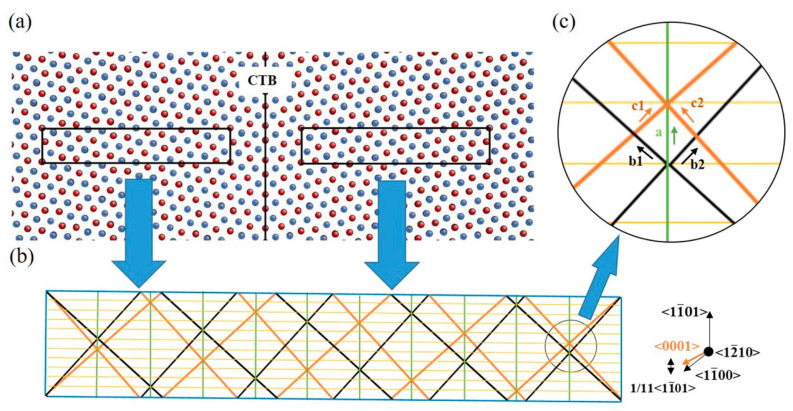
(**a**) Simulation box containing $\{1\overset{-}{1}02\}$ CTB with the red and blue dots indicating atoms on the first and second layers, respectively; (**b**) Schematic illustration of DSC lattice of $<1\overset{-}{2}10>$Σ11 supercell of α-Ti, black, orange, green, yellow lines mean two symmetrical lattices and longitude, latitude lines, respectively; the circled region is zoomed in (**c**).

Atomic-scale simulations were conducted using LAMMPS [20] and the embedded atom method (EAM) potential [21] for Ti. This interatomic potential has been widely utilized in previous investigations and has proven to be suitable for studying the mechanical behaviors of Ti [19,22,23,24,25,26]. To model the mutual transformation between CTB and BPB, a bi-crystal model with the $\{1\overset{-}{1}02\}$ CTB [27] (Figure 1a) was constructed using ATOMSK [28], according to the experimentally observed microstructure in Ti and Mg [3,4,5]. The simulation box had dimensions of 24.3 nm × 5.9 nm × 60.8 nm with 492,800 atoms under fully periodic boundary conditions. The system was initially statically relaxed with an energy criterion of 10^−6^ eV and then underwent relaxation at 1 K for 20 ps to reach a stable grain boundary structure. Subsequently, shear loading was applied along $<10\overset{-}{1}1>$ with a constant strain rate of 10^8^/s. All MD simulations were taken with a timestep of 10^−12^ s. Atomic configurations were colored and visualized using AtomEye [29].

## 3. Results

### 3.1. Accumulation of TB Steps

**Figure 2:**
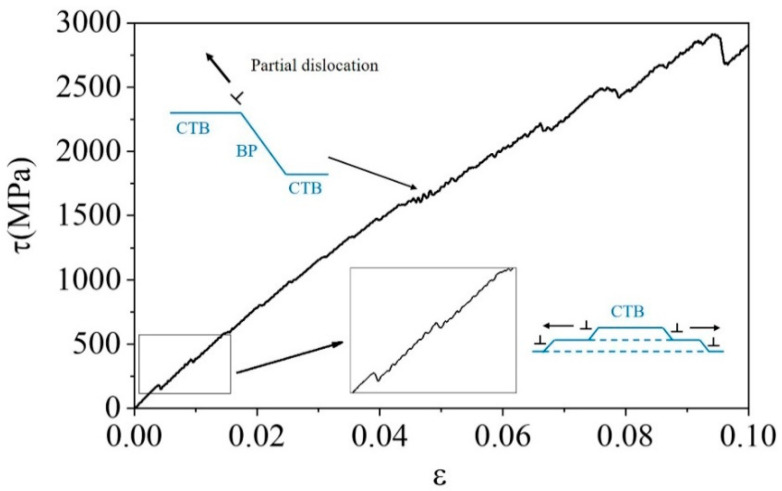
Shear stress–strain curve and schematic diagrams of CTB growth. Arrows near the dislocation symbol refer to their gliding directions.

**Figure 3:**
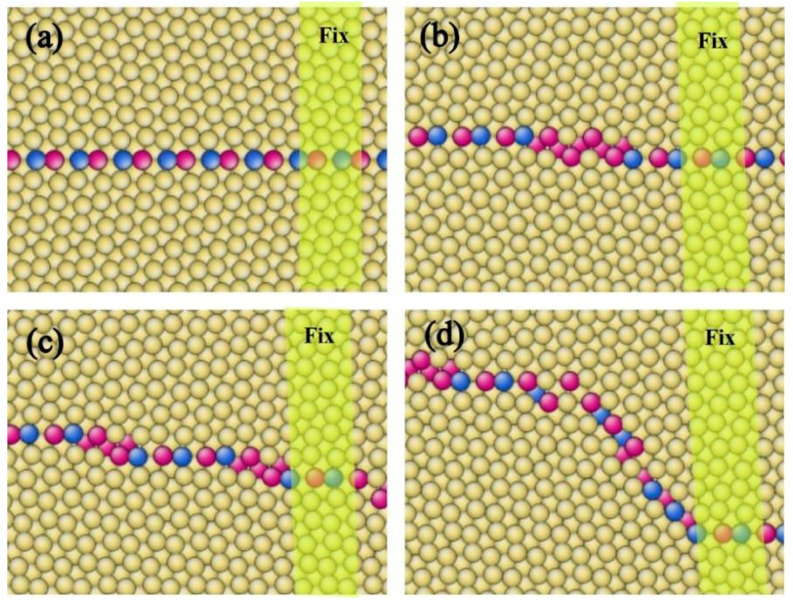
Gathering of TB steps and formation of BPB at different shear stress on one side. Colored by coordination number (**a**) ε = 0 (**b**) ε = 0.015 (**c**) ε = 0.03 (**d**) ε = 0.045. Atoms were colored according to their coordination number.

The stress–strain curve during shear loading is presented in Figure 2. The first peak appears on the curve at a strain of approximately 0.5% and a stress of approximately 200 MPa. Consequently, a pair of TB steps nucleate and propagate in the opposite direction, as depicted in Figure 3a–c. This process repeats at every stress increment of 200 MPa or strain increment of 0.5%. The lattice orientation of a layer of atoms changes upon the appearance and blockage of a pair of steps. With displacement shift complete (DSC) vectors analysis (Figure 1b), a single layer step on the CTB, denoted as 1d (‘d’ refers to the unit of the step.), cannot be formed without any displacement in the matrix lattice, and thus a disconnection with Burgers vector of $\frac{1}{11}<1\overset{-}{1}01>$ is required, as shown in the green vector in Figure 1c. It should be noted that this disconnection can decompose in the direction of vectors b and c presented in Figure 1c, which are nearly perpendicular to each other according to the small angle approximation.

(1) $$\frac{1}{11}<1\overset{-}{1}01>\to \frac{1}{11}<\overset{-}{1}100>+\frac{1}{11}<0001>$$

**Figure 4:**
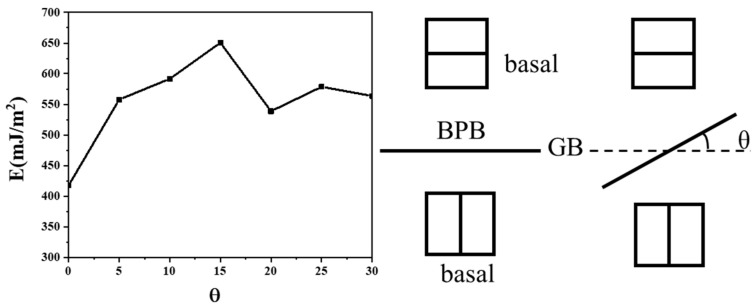
Boundary energies of different boundary orientations for GB initiated from BPB with grain misorientation 90° rotated around $<1\overset{-}{2}10>$ axis.

This disconnection also induces lattice transfer between the <0001> and directions, which are perpendicular to the basal and prismatic planes, respectively. This suggests that the disconnection is necessary for the formation of BPB, especially by component b1 or b2 in Figure 1b, with b2 providing prismatic planes for the matrix grain in this case. As illustrated in Figure 4, BPB has a boundary energy of approximately 417 mJ/m^2^ with some remaining disconnections on the boundary, slightly higher than the CTB, which has a boundary energy of approximately 278 mJ/m^2^. The energy on BPB may be lower when the boundary is short and meets the coherent condition. Compared to general boundary orientations where θ is not zero in Figure 4, BPB is a stable structure that can be easily formed with a much lower boundary energy close to CTB. Blocked steps are arranged in a line with some distances at low strain for the same sign of Burger vectors, as shown in Figure 3b, but the distance reduces when new steps appear and eventually merge into a step with a height of 2d, similar to BPB. This phenomenon is also reported in the simulation of Mg [30]. With more steps appearing and blocking, the height of the steps steadily grows into the structure shown in Figure 3d.

As the height of the steps increased to 8d, the stress within the system rose to 1600 MPa, causing a small fluctuation in the shear–stress curve, as shown in Figure 2. At this point, the interfaces on different sides exhibited distinct behaviors, and a partial dislocation was observed to emit from one side of the boundary.

### 3.2. Emission of Shockley Partial Dislocation

**Figure 5:**
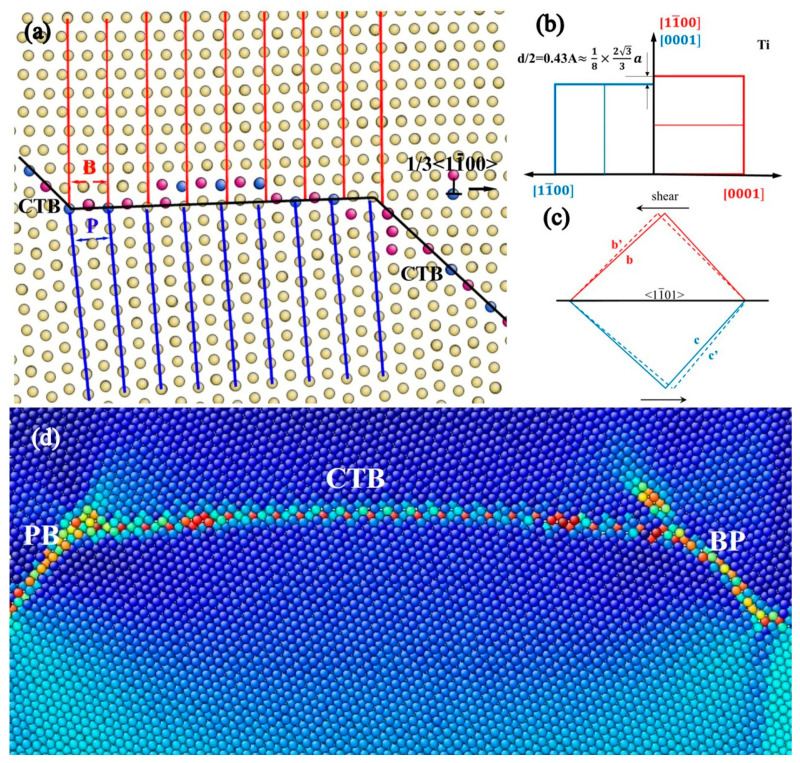
Schematic diagrams of mechanism of partial dislocations escape from BPB. (**a**) Snapshot of emitting of partial dislocation (**b**) Lattice mismatch of BPB in Ti. (**c**) Schematic illustration of lattice distortion at shear strain ε (**d**) atomic structure of boundaries colored by relative shear stress, atoms colored by dark blue represent lower shear stress. Atoms were colored according to their coordination number and local von Mises stress in (**a**) and (**c**), respectively.

When the shear strain reaches 4.6%, Shockley partial dislocations are emitted from the boundary with its upper grain providing basal plane and the lower providing prismatic plane, as shown in Figure 5a. This phenomenon can be roughly explained by morphology. As mentioned in the introduction, most HCP metals deviate from a perfect HCP structure, resulting in a slight misfit between the lattice parameters of the basal and prismatic planes, as shown in Figure 5b, which leads to elastic energy stored in coherent BPBs. A wide BPB is unstable with the accumulation of elastic energy, which can be released by inserting a plane of prismatic. For example, HCP-Ti has 12 basal planes on one side of boundaries and 13 prismatic planes on the other, while the misfit of incoherent BPB would decrease to less than 0.5%. Most simulations in Mg use this method to stabilize the BPB [3,4,31]. However, it is challenging to obtain such a structure during mechanical deformation. When the edge BPB links with CTB, some events should occur to offset the extra energy immediately when the incoherent interfaces appear. Basal stacking faults with $\frac{1}{3}<1\overset{-}{1}00>$ partial dislocations, which can be easily formed in HCP metals, can play the same role in relaxing elastic stress as inserting an additional prismatic plane to keep the boundaries coherent. It is easiest for partial dislocation emission when the deformation of the basal plane is nearly equal to the Burger vector of the partial dislocation, with the assumption that the deformation of the basal and prismatic planes is approximately the same. To meet the condition of coherency, the number of layers of the basal plane n should minimize the equation $(n+\frac{1}{3})b=(n+1)c$, where b and c are lattice parameters. In this way, $\frac{1}{3}<1\overset{-}{1}00>$ Shockley partial dislocations should be emitted from the tip of BPB and remain a mismatch of $\frac{1}{3}<1\overset{-}{1}00>$. The value of n in Ti is 8, which means that a partial dislocation should emit at every eight layers of BPB but cannot move too far from the boundary due to the limitation imposed by the coherent condition. The results obtained in Figure 5a are consistent with our analysis. Moreover, if the same result is found in Mg, the value of n should be 11, but it would be greatly influenced by the stacking fault energy.

We observed that in the lower grain shown in Figure 5d, atoms near the BPB experienced shear stress compared to those in the matrix, which can be attributed to lattice distortion. As depicted in Figure 5c, at a shear strain *ε*, the lattice parameter b of the upper grain must be modified to

(2) $$b^{\prime }=(\frac{(b^{2}-\varepsilon bc{)}^{2}+b^{2}c^{2}}{b^{2}+c^{2}}{)}^{\frac{1}{2}}$$

And analogously, *c* of lower grain should be modified to

(3) $$c^{\prime }=(\frac{(c^{2}-\varepsilon bc{)}^{2}+b^{2}c^{2}}{b^{2}+c^{2}}{)}^{\frac{1}{2}}$$

The modifiers exert a similar influence on both upper *b* and lower *c*, while *b*′ and *c*′ decrease from 5.16 Å and 4.73 Å to 5.00 Å and 4.58 Å, respectively. However, the value of *b*′ should descend more at BPB, which impedes the further deformation of the basal plane. For the prismatic plane, the BPB offsets some shear stress, and the orientation of the newborn twinning grain is closer to the state without any stress. In other words, it is mainly the prismatic planes that adjust their size to fit the BPB. It can also be observed that the BPB is not flat, which is consistent with the results calculated and predicted by Barrette et al. in Mg [32].

### 3.3. Repetition of the Accumulation/Emission Process

**Figure 6:**
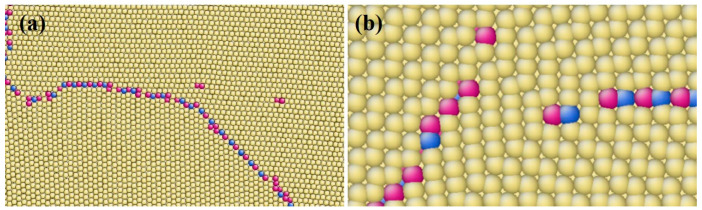
(**a**) Emitting of second partial dislocation, while twin step is about 16d (**b**) Areas of stacking faults at PBB of boundary. Atoms were colored according to their coordination number.

With the application of a shear strain, the BPB grew along the route of a partial dislocation, and a layer of FCC structure remained on the boundary between the two grains. Compared with the BPB in the HCP structure, this boundary is more similar to the phase boundary between the HCP and FCC structures with an abnormal orientation relation. New partial dislocations were emitted when the height of the TB steps grew to another 8d, which enlarged the area of the stacking fault and lengthened the GB, as shown in Figure 6a. With subsequent loading, more than 20 layers of GBs can be observed, but only eight layers of them can be seen as perfect BPBs in the HCP structure, while the others, though different from BPBs and more similar to phase boundaries, may be difficult to recognize only by structural analysis under an electron microscope.

With the subsequent applied strain, the TB began to climb along a fixed area, indicating that it was more difficult to create new steps than to overcome the hindrance of the fixed area. When the shear strain reached about 9%, the lattice parameters of the matrix grains b′ and c′ were the same, according to Figure 5c and Equations (2) and (3). Meanwhile, the first minus sign in Equation (3) should be replaced with a plus sign, resulting in a new twin nucleus from the matrix grain. The BPB could not continue growing with the accumulation of steps, but there remains the possibility that the BPB can be enlarged by getting BPBs from different CTBs connecting with each other.

As for another group of blocked steps with a reverse moving direction, steps gathered and formed boundaries that should be called prismatic–basal boundaries (PBBs) to distinguish them from the BPB on the other side. In this area, the upper grain provides the prismatic plane and the lower grain provides the basal plane, which is contrary to the BPB mentioned earlier. Partial dislocations did not escape from the tip of the PBB when this phenomenon happened on the BPB because of the block of the fixed area and the unfavorable stress states. Instead, the partial dislocations would move vertically to the PBB with the same direction as that from the BPB and leave a layer of conical stacking fault, as shown in Figure 6b, which divided the two grains with much lower local stress than that on the CTB or BPB. According to traditional theory, phase transformation from HCP to FCC by stacking faults has orientation relationships of <0001>_HCP_||<111>_FCC_, where both of them are close-packed planes of their own structure. While the angle between <111> and $<\overset{-}{1}11>$ directions in the FCC structure is about 70.5°, FCC phases nucleated from different sides of the CTB have a misorientation of 14.5°. This deviation can be adjusted by elastic deformation and some stacking faults with lower local shear stress and energy increase. However, it should be mentioned that the potential file used in the simulation has a much lower stacking fault energy compared to the value obtained from experiments [33], which can severely influence the results of the simulation and increase the chance of partial dislocations appearing. Nevertheless, this structure with a large number of stacking faults may serve as an effective way to connect grains with large misorientation.

## 4. Discussion

Previous findings suggest that the imbalance between the movement of $\{1\overset{-}{1}02\}$ CTBs and the accumulation of TB steps may play a crucial role in the transformation of CTBs and BPBs in titanium. Defects such as dislocations, micro-cracks, and grain boundaries can take on the role of fixed atoms. Grain defects can cause an imbalance in movement and facilitate the appearance of BPBs. For example, Serra et al. [33], Barrett et al. [32], and Li et al. [34] found BPBs in alpha titanium and Mg nanopillars by MD simulation, respectively. Moreover, most BPBs found in Mg alloys by microscopes are located at the tip of twin boundaries, where high stress exists [35,36].

**Figure 7:**
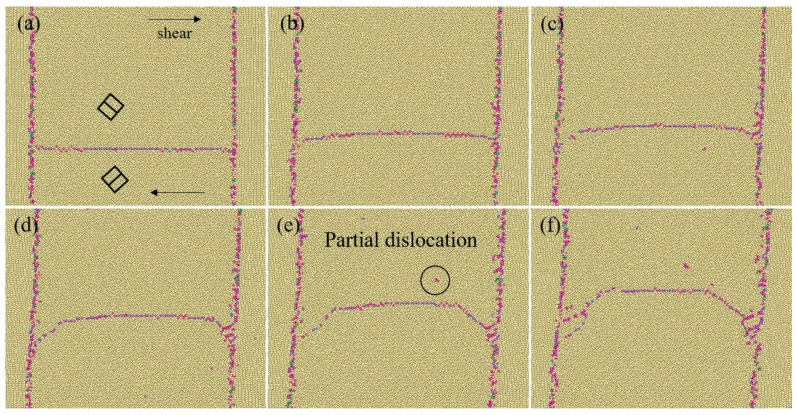
Moving of $\{10\overset{-}{1}2\}$ CTB with vertical boundaries during shear loading at 300 K (**a**) ε = 0, (**b**) ε = 0.02, (**c**) ε = 0.03, (**d**) ε = 0.04, (**e**) ε = 0.05, and partial dislocations emitted, (**f**) ε = 0.06. Atoms were colored according to their coordination number.

To simulate this phenomenon, we replaced the fixed area with a grain with its c-axis perpendicular to the CTB, chosen for convenience, though the new grain can be of random orientation (Figure 7). The model size was adjusted to maintain full-period boundary conditions. Upon shear loading, the CTB-BPB transformation at the two endings of the CTB also results in steps with opposite signs. Such steps can move along the CTB and annihilate, which moves the CTB upwards. When a suitable number of steps was gathered, a Shockley partial was emitted. We observed similar behaviors in the model with the general boundary and the flat CTB as we did in the model with a fixed area, such as the appearance of BPBs and the emission of partial dislocations. The results were the same at 1 K, but at room temperature, TB steps could not be clearly identified, and defects in general boundaries affected the structure of the root of boundaries. Upon unloading the strain, the CTB with the fixed area returned to its original position after absorbing partial dislocation, while the CTB with general boundaries deviated slightly from its initial position. The insertion of a micro-crack or other defects led to similar results, where boundaries changed into a trapezoidal shape or bypassed defects with BPBs as transition structures.

**Figure 8:**
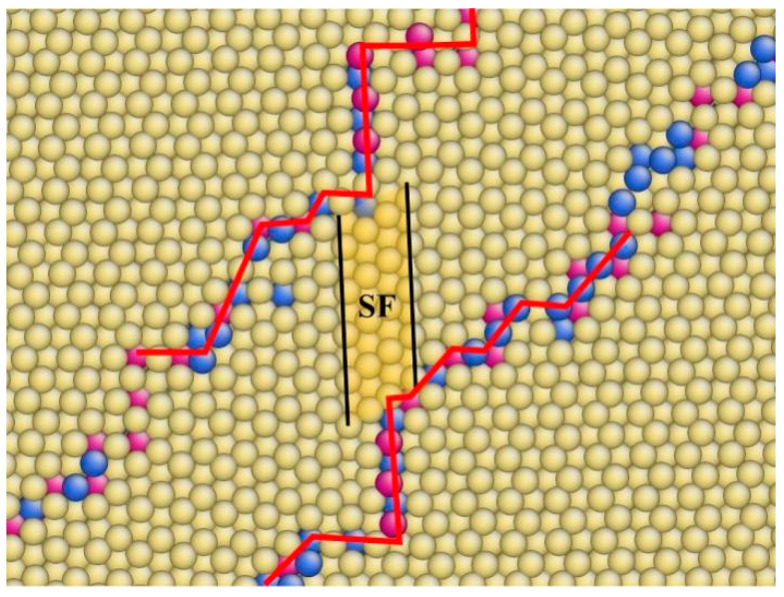
Stacking fault and distortion of $\{1\overset{-}{1}02\}$ CTB close to another one after partial dislocation emitted from the upper boundary and absorbed by lower one. Atoms were colored according to their coordination number.

We believe that $\frac{1}{3}<1\overset{-}{1}00>$ partial dislocations play a key role in the transformation of CTBs by providing extra Burgers vectors and relieving stress in the system. Other researchers have also found that when normal strain is applied to the BPB of Mg, it produces CTBs with remaining partial dislocations [4,31]. Partial dislocations not only offset the disconnection between basal and prismatic planes but also provide disconnections for CTBs to form BPBs by interacting with boundaries, indicating that partial dislocations may act as intermediates between these two boundaries. Large-angle boundaries can change forms by emitting or absorbing partial dislocations. Li et al. reported on the movement of CTBs without shear, in which case, partial dislocations are difficult to emit due to small Schmidt factors and weak hindrance [34]. However, if a model with two layers of CTBs close to each other is subjected to a sufficiently large normal stress, CTBs can be distorted by thermal vibration and complex stress fields, and partial dislocations can be emitted, leaving behind a stacking fault. These partial dislocations can then be absorbed by the other side, causing further distortion, as shown in Figure 8. A wider BPB can be observed at the tip of the stacking fault. The normal direction of the boundaries after the partial dislocation emission and absorption is about 45° deviated from the CTB, nearly the same as the boundary orientation of the BPB. The absorption of partial dislocations in CTBs can be roughly formulated with some approximation as follows:

(4) $$\frac{1}{3}<1\overset{-}{1}01>+\frac{1}{3}<\overset{-}{1}100>\to \frac{1}{3}<0001>$$

The formula can be interpreted as follows: The first item on the left of the formula represents four layers of twin boundaries, the second item represents a partial dislocation, and the item on the right represents four layers of BPB, suggesting that a piece of BPB with a length of 4d can be formed on the boundary after interacting with a 1/3 partial dislocation. Similarly, an inverse PBB can be identified after partial dislocations are emitted from CTBs; however, this approach ignores the coherent condition, and a piece of PB/BP interface should accompany the new-born BP/PB interface, producing a large number of partial dislocations near boundaries. Numerous partial dislocations have been reported in experiments to accompany CTBs, not only in titanium [37]. These partial dislocations can serve as intermediates of transformation or the final products after transformation.

Partial dislocations escape under conditions of high stress. The nucleation of twins always begins with the highest stress level, accompanied by a complex stress field and drastic fluctuations in structures, making it easy to emit partial dislocations and causing boundary distortions. For short distances of CTBs, partial dislocations can also be absorbed by the other side, resulting in a series of stacking faults in twin grains. Stacking faults will not disappear during the growth of twins, but the partial dislocation nuclei will move with the CTB and expand the area of stacking faults, leading to a large number of faults inside the twin grains.

Lastly, it is worth briefly comparing the simulated GB structure with the experimental observations. For HCP-Ti, as indicated in Figure 1b of Ref. [36], the boundary exhibits interconnected CTB and BPB exactly as in Figure 1 and Figure 7 in the present simulation, while for HCP-Mg, a similar boundary structure appears in Figure 2 and Figure 4 of Ref. [4]. Regarding the profuse existence of stacking faults, the present work proposes a possible route that may help to explain the observations in Figure 9 of Ref. [38]. The present results regard the transformation of GB under external loading, and they can thus be applied to wrought Ti and Mg alloys to help regulate their microstructure for property enhancement or processing optimization.

## 5. Conclusions

MD simulations indicate that the transformation between $\{1\overset{-}{1}02\}$ coherent twin boundary (CTB) and basal–prismatic boundary (BPB) in HCP-Ti occurs under external loading. The main findings are summarized as follows:

(1) Under external loading, CTB undergoes transformation into BPB through the accumulation of TB steps and subsequent emission of Shockley partial dislocations. Such transformation occurs gradually, as successive step accumulations and partial emissions adjust for the lattice mismatch between the basal and prismatic orientations.
(2) Shockley partial dislocations play a critical role in the transformation process. When the total mismatch vector is equal to or close to that of a Shockley partial dislocation, BPBs emit partial dislocations and further grow along the stacking faults.
(3) When a pair of CTBs are close to each other, the complex stress state and drastic fluctuations result in severe boundary distortions, facilitating the emission and absorption of partial dislocations, which further assists the transformation from CTB to BPB.

## Data Availability

Data are contained within the article.
